# Activity monitoring of stroke patients by physiotherapist and caregivers in a hospital setting

**DOI:** 10.12688/f1000research.124675.1

**Published:** 2022-10-27

**Authors:** Apoorva M. Shankaranarayana, Yakub Sameerkhan Pattan, Nikhil Hegde, Manikandan Natarajan, Aparna R. Pai, Raghavendra Nayak, John M. Solomon

**Affiliations:** 1 Department of Physiotherapy, Manipal College of Health Professions, Manipal Academy of Higher Education, Manipal, Karnataka, 576104, India; 2 Centre for Comprehensive Stroke Rehabilitation and Research, Manipal College of Health Professions, Manipal Academy of Higher Education, Manipal, Karnataka, 576104, India; 3 Department of Neurology, Kasturba Medical College Hospital, Manipal Academy of Higher Education, Manipal, Karnataka, 576104, India; 4 Department of Neurosurgery, Kasturba Medical College Hospital, Manipal Academy of Higher Education, Manipal, Karnataka, 576104, India

**Keywords:** Behavioural mapping, activity monitoring, stroke, hospitals, caregivers

## Abstract

**Background:** Activity monitoring is a necessary technique to ensure stroke survivors’ activity levels in the hospital are within optimal levels as this is important for enhanced motor recovery. However, this could be time-consuming for healthcare professionals like physiotherapists. Activity monitoring by caregivers could be an alternate option. Therefore, our aim was to compare the activity monitoring of stroke survivors by caregivers and physiotherapists in a hospital setting.

**Methods:** An observation study was carried out in the neuroscience ward in a tertiary care hospital among 17 stroke survivors. Physiotherapist and caregivers were instructed to use an activity log chart that was developed during previous research conducted by the same authors for observing the activities performed by the patients every 15 minutes from 8 AM to 5 PM. Data collected were analysed using Stata 15. Kappa statistics were carried out to determine the agreement of the observations between the two raters.

**Results:** A total of 10 male and seven female caregivers of stroke survivors with a mean age of 40.11 ± 9.2 years participated in the study. A total of 272 observations of caregivers were in agreement with that of the physiotherapist. Inter-rater Kappa statistics showed 60% agreement between the physiotherapist and the caregivers, while the multi-rater Kappa for different time points did not show agreement (Kappa value <0.1).

**Conclusions:** There was moderate agreement between the physiotherapist and caregiver for activity monitoring of stroke survivors. This suggests behavioural mapping by caregivers may be a potential alternative solution in healthcare settings.

## Introduction

Stroke is one of the leading causes of death and disability worldwide. ^1^ The American Heart Association has predicted that 2.58 million people will have suffered a stroke by 2047 in Europe alone. ^2^ Functional impairment following acute illnesses, such as stroke, frequently has negative consequences, including sensory, motor, psychosocial, cognitive, and sexual dysfunctions. ^3^^–^^6^ Due to these impairments, stroke survivors have significantly reduced activity levels from an early phase. ^7^

The importance of being active from an early phase is well-established in stroke survivors. The evidence for functional recovery is rapid in the acute phase and depends on several factors including the amount of activity that is done by the patient. ^8^^,^^9^ Hence, it is vital for stroke survivors to be active from an early phase. However, patients undergoing inpatient rehabilitation after stroke have limited opportunities to be active. ^10^^,^^11^ As a result, sedentary behaviour during the hospital stay could limit the potential for optimal stroke recovery. Studies suggest that stroke survivors are sedentary in hospital settings and are described as ‘inactive and alone’. ^9^ This is concerning because of the strong association between higher levels of inactivity and a decreased rate of functional recovery. ^12^ For this reason, special interest has been placed to explore the amount of activities in stroke patients during the early phase. ^13^

Behavioural mapping is a well-known observational method that can be used for recording and observing various behaviours. ^14^ It allows researchers and clinicians the opportunity to collect, analyse and represent information in resourceful ways, which help to determine how one's environment may influence their behaviour. ^15^ It is an effective tool to represent behavioural patterns in any location. ^16^^,^^17^ Behavioural mapping has also been used for assessing patients' behaviour in hospital settings, including monitoring their physical, cognitive and social activities. ^18^^–^^20^

By using this method to measure stroke survivors’ activity levels in hospital wards can help determine their activity and sedentary behaviour. Existing studies show that behavioural mapping for activity monitoring is mostly carried out by professionals or researchers, including physiotherapists, and is usually done either for one or multiple days. ^18^^,^^19^^,^^21^^–^^23^ However, this method poses a challenge for the healthcare workers as it can be time-consuming due to a longer evaluation period. Hence, it may require multiple people to monitor the activities of the patient, making the method less feasible.

Activity monitoring by caregivers of the patients may be an alternative solution. Caregivers are known to be with the patient for a large amount of time during their hospital stay. ^24^ However, the accuracy of measurement by caregivers compared to monitoring by rehabilitation professionals need to be ascertained. Therefore, the study aimed to determine the interrater agreement between the activity monitoring of stroke survivors carried out by the caregivers and physiotherapist in an acute hospital setting.

## Methods

### Ethics

This study was a part of a larger ongoing study that aims to enhance the activity levels of stroke survivors and was approved by the Institutional Ethics Committee, Kasturba Medical College and Kasturba Hospital, Manipal (IEC 438/2019) on 16 ^th^ July, 2019. This study was conducted prior to the commencement of the main study and included a different population of participants. The study was conducted in the neuroscience ward of Kasturba Hospital, Manipal in Southern India.

### Participants

*Eligibility criteria*

All the stroke patients admitted to the ward were screened for the eligibility criteria from July 2020 to November 2020. As this was a pilot study, we conducted a time-bound design. We included caregivers of stroke patients affected with the supra-tentorial lesions, aged between 18 and 80 years, medically stable with no other comorbidities and who could functionally communicate. We excluded caregivers of patients who underwent surgery and with other impairments like fractures, musculoskeletal, cardiovascular, neurological and other chronic diseases that could affect their activity levels. In addition, we only included primary caregivers who are with the patient for most time during the day in the ward. Further, we included caregivers without any psychological/psychiatric disorders and who could functionally communicate.

### Outcome

The behavioural mapping was carried out using an activity log chart that has been developed to monitor the activities of the stroke patient during their hospital stay. It has components of physical, cognitive and social activities that stroke patients perform in a hospital. All the activities in the activity log chart were finalized after conducting a thorough literature search and observations of activities performed by the stroke patients in the hospital for nine hours per day for a duration of one week. The log chart has activities written in English and Kannada (regional language) along with the image depicting the activity being performed. This was to ensure that the caregivers comprehend the log chart, irrespective of their education level. The activity log chart can be found as *Underlying data.* ^31^ Further, the log chart was sent to 15 experts for validation and was tested on 20 stroke patients prior to the commencement of this study (unpublished work, Shankaranarayana AM, Natarajan M, Solomon JM). The copyright for the log chart has been applied with the Government of India.

### Procedure

All stroke patients admitted in the neuroscience ward were screened for the criteria. Eligible patients and their caregivers were explained about the study, and written informed consent was obtained from both patients and caregivers. An intern in physiotherapy was recruited and informed consent from the physiotherapist was taken. The caregivers and the physiotherapist were explained about the procedure of monitoring the patients along with the usage of the activity log chart. All the instructions to record the activities were provided one day prior to the day of observation and the principal investigator clarified any queries regarding activities to be monitored. In addition, the caregivers were also trained to use the activity log chart by simulating examples.

The activities in the chart were grouped into physical, social and cognitive activity, and both the raters (caregiver and the physiotherapist) were asked to mark a tick (✓) against the corresponding activity that the patient was doing at a particular time slot. At any time, if the patient was doing two or more activities at the same time (example: eating and reading, walking and talking), the raters were asked to mark both the activities. The observations were carried out every 15 minutes by both the caregiver and physiotherapist for a single day. This allows a total possibility of 37 observations per patient by each rater. The principal investigator (AMS) provided the chart before 8 AM on the day of observation and collected it back at 5 PM after the caregiver completed all the observations. Both the caregiver and physiotherapist were instructed and monitored by the principal investigator to not discuss or see the other person’s chart to prevent contamination of results. The caregivers were informed to monitor the patients’ activities as much as they possibly could during that time period. A research assistant was consigned to conduct periodic monitoring with the caregivers about their activity monitoring.

### Statistical analysis

Descriptive statistics were used to summarize the demographic characteristics of patients and caregivers. Percentage agreement for monitoring between the caregiver and the physiotherapist was calculated for overall activities, each domain and each activity. Analysis was carried out using Stata 15 (RRID:SCR_012763) (free alternative, Rstudio). Agreement between the two raters domain wise was assessed using Kappa statistics. Multi-rater kappa was used to assess the agreement across the different time points. Kappa values of ≤ 0 as indicating no agreement and 0.01–0.20 as none to slight, 0.21–0.40 as fair, 0.41– 0.60 as moderate, 0.61–0.80 as substantial, and 0.81–1.00 as good agreement. ^25^

## Results

A total of 60 stroke participants were assessed for eligibility and 17 were recruited for this study. The main reasons for excluding were patients who underwent surgery (n=26), patients with recurrent stroke (n=12) and those who could not comprehend (n=5). The demographic characteristics of the stroke survivors and caregivers who participated in the study are given in Table 1 and Table 2, respectively. ^31^

**Table 1.** Demographic characteristics of the stroke survivors in the study (n=17).

Characteristics	Value
Age in years (Mean ± SD)	53.35 ± 16.4
Sex	
Male:	10
Female	07
NIHSS score	
Mild:	11
Moderate:	04
Moderate to severe:	02
Post stroke duration in days (Mean ± SD)	3.47 ± 1.32
Type of stroke	
Ischaemic:	11
Haemorrhagic:	06
Side of stroke	
Left:	12
Right:	05

NIHSS, National Institutes of Health Stroke Scale.

**Table 2.** Socio-educational details of the caregivers who participated in the study (n=17).

Characteristics	Frequency (number of participants)
Relation to the patient	
Spouse:	08
Children/Grandchildren:	06
Daughter-in-law:	01
Sibling:	02
Education	
Nil:	01
<High school:	04
High school:	08
Graduate:	04
Occupation	
Homemaker:	05
Farmer:	03
Employed:	05
Healthcare worker:	01
Own business/freelancer:	03
Basic knowledge * about stroke	06
Previous experience as a caregiver in a hospital	05

* Fundamental causes, treatments and prognosis of the stroke.

A total of 17 caregivers of the stroke survivors, of which 10 were male and seven female participated in the study with the mean (SD) age of 40.11 ± 9.2 years. The socio-educational details of the caregivers who participated in the study are given in Table 2.

The behavioural mapping carried out showed the following observations. Out of the possible 629 observations (37 observations/participant), the physiotherapist marked 535 (85%), while caregivers marked 424 (67.5%). A total of 272 out of 424 (64.2%) caregiver observations had an agreement with the physiotherapist observations.

**Figure 1.**
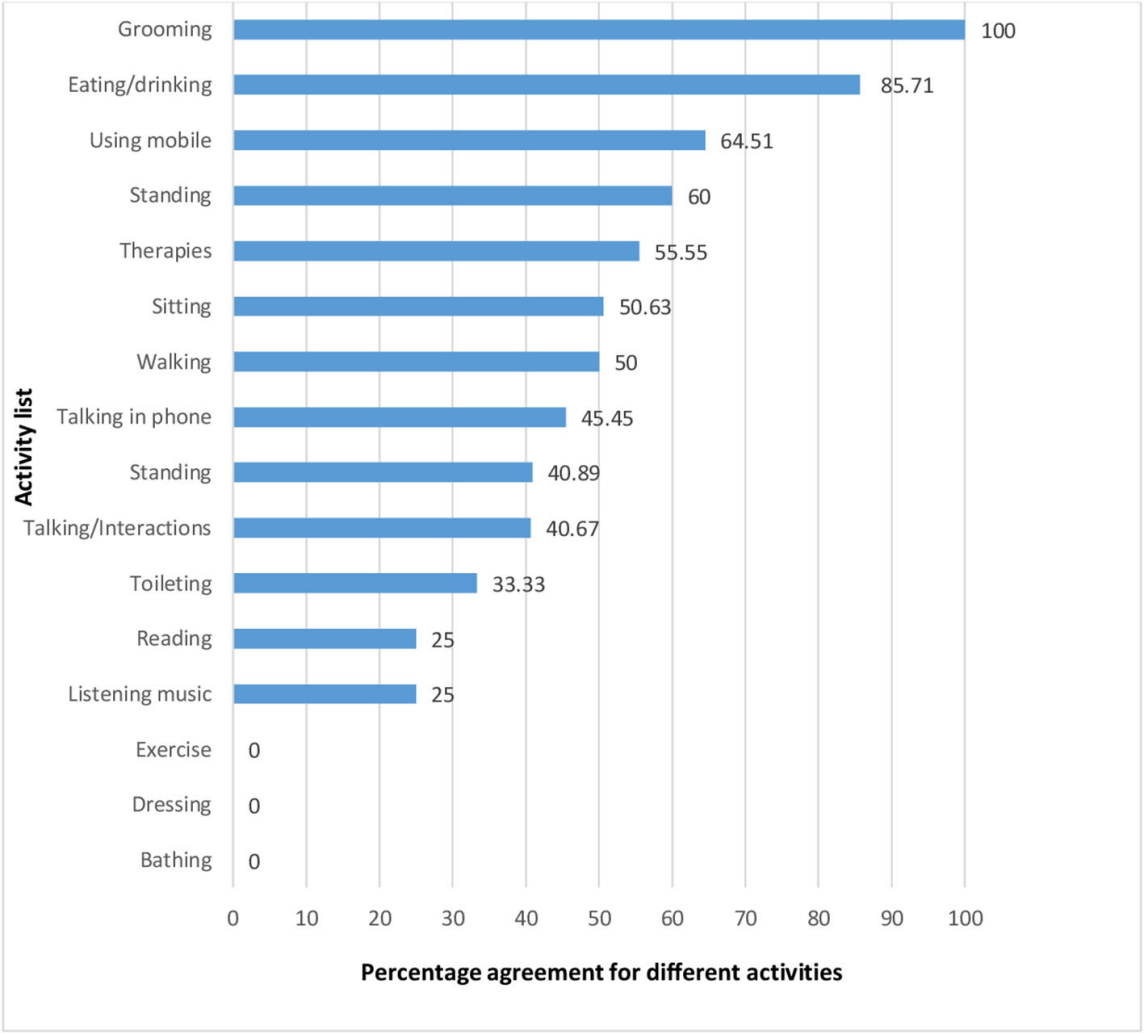
Percentage agreement for different activities.

Agreement between caregivers and physiotherapist varied significantly for different activities as it ranged from 0% (lowest) for bathing, dressing and 100% (highest) for grooming. The percentage agreement for different activities monitored by caregivers and physiotherapist are shown in Figure 1. Further, the agreement of activities under physical, cognitive and social domains were 42, 38 and 43%, respectively.

Inter-rater agreement between the caregiver and the physiotherapist for the various activities showed a Cohen’s Kappa of 0.61 with 95% CI (0.55, 0.66) and p value <0.001, while the multi-rater Kappa for different time points in a day showed <0.1 agreement.

## Discussion

The aim of this study was to compare the observations made by physiotherapist and caregivers to capture the possibility of the caregivers in monitoring the patients’ activities in acute care settings. Our results showed that the agreement between both the observations was 64.2%, implying that caregivers could not monitor the activity of patients as accurately as the physiotherapist. However, the discrepancy in the observations could be due to many reasons. First, the subjectivity of the behavioural mapping by itself could have led to the variability in observations. ^26^ Second, though the instructions were given to observe every 15 minutes, the time of observation could have varied by seconds between the physiotherapist and the caregivers, which is enough to change the activity. For example, in the initial seconds of 8 AM (8:00:00) the patient may have been sitting, but by the end of 8 AM (8:00:45), he may be standing and talking. Hence, the patient could have switched their activity between those two observations, leading to variability. Another reason may be due to the variability that could have occurred in situations while the patient was performing more than one activity simultaneously. Although the caregivers were asked to mark all the activities in such situations, it was noted that quite a few times the caregiver had only marked a single activity whilst the physiotherapist had marked dual activities. For instance, a patient walking while talking over the phone was marked for walking alone by the caregiver, while the physiotherapist marked both walking and talking on the phone.

Though the caregivers are with patients most of the time in the hospital, they may move out of the ward for various requirements related to the patient and for other personal reasons. These reasons could explain the reduced percentage of observations by caregivers compared to the physiotherapist. These reasons were supported by a recent study, which states that a family caregiver has high intensity role in the hospital as they have to multitask both physically and mentally. ^27^ These roles make it challenging for them to tend to additional work besides situations associated with illness and dependency of the patient during the hospital stay. ^28^ Additionally, evidence shows that caregivers of acute diseases like stroke have more compounded situations due to the sudden change in adaptation required compared to chronic diseases. ^29^ Another reason could be the change in caregiver of the patient during the observation day. The replaced caregiver would have not received the entire instructions from the previous caregiver leading to loss of vital information regarding the observations. Hence, the new caregiver might not have understood the procedure adequately and did not record the activities diligently. Further, although we used pictures along with words to depict the activities in the log chart, we noticed that many caregivers had not marked the activities for all the time slots. The comprehension level and differences in the education level could be the reason for this. All caregivers had some level of formal education except for one.

A total of 12 caregivers in our study had no previous hospital experience. Since the majority of the caregivers lacked experience in managing a hospital, anxiety and unfamiliarity of the situations in the hospital could have been the reason for the overall reduced activity loggings. This was supported by an earlier study that showed that new caregivers have a higher level of burden and anxiety in the hospital, ^24^ which might have influenced the observations significantly.

We noticed that caregivers could log some additional activities that the physiotherapist could not. Bathing and dressing were a few such activities that the physiotherapist had not marked. Due to the separate bathing area where the caregivers accompanied the patients sometimes to assist, they could log the activity. However, the physiotherapist on such occasions, could not differentiate and had either not logged anything or marked it as toileting. Whereas, overall, the caregiver had logged both toileting and bathing appropriate to the time slots. In this study, both the physiotherapist and the caregiver did not complete all observations. In the hospitals, for various tests, patients are taken to different test/diagnostic rooms, ^30^ during which, it would be difficult to monitor patients for their activities. This could be one of the primary reasons for lesser observations made by both physiotherapist and caregivers.

The Kappa statistics showed 60% agreement between the physiotherapist and the caregiver. Even though this is not ideal, there is moderate level agreement seen. There may be a potential for enhancing the agreement levels if all the above-mentioned problems are addressed. However, we had a low agreement across the time points on multi-rater kappa. This could be due to the low sample for each time point across all the patients.

To our knowledge, this is the first study that compared behavioural mapping between the physiotherapist and the caregivers for the activities performed by stroke patients. The limitation of this study is that it had a low sample size, as this was a phase in a bigger study. We did not do location mapping during the behavioural mapping as it was a fixed location. All the patients recruited were from the same general ward. In addition, as the monitoring was new and unfamiliar to the caregivers, we did not impose the extra detail of location and people present, which are usually carried out during behavioural mapping. Second, the observations in the study were done only on a single day. Third, behavioural mapping is a subjective measure of assessment. However, it is the best available method for assessing or recording an individual’s behaviour.

Although activity monitoring by caregivers was in moderate agreement with the observations made by physiotherapist, it is important to note that some of the toiletry activities were monitored only by the caregivers. Further, the agreement level may have scope for improvement considering that some of the above issues are modifiable. Thus, there is a potential for caregivers to perform behavioural mapping of stroke. This paves way for a feasible method of behavioural mapping in healthcare settings. Future studies are directed towards the larger sample and longer periods of activity monitoring.

## Data Availability

Figshare: F1000 data final. https://doi.org/10.6084/m9.figshare.21076363. ^31^ This project contains the following underlying data:
- Activity Log Chart.pdf - Data repository.xlsx (participant spreadsheet data) Data are available under the terms of the Creative Commons Attribution 4.0 International license (CC-BY 4.0).
