# Supplementary material for: Activity monitoring of stroke patients by physiotherapist and caregivers in a hospital setting: A pilot study
Source: F1000Res. 2023 Nov 10;11:1227. Originally published 2022 Oct 27. [Version 2] doi: 10.12688/f1000research.124675.2 (PMC10638484; doi:10.12688/f1000research.124675.2)
Supplement: Peer review history

# Reviewer report: Reviewer response for version 2

**DOI:** 10.5256/f1000research.158690.r222341

**Published:** 2023-11-10

**Authors:** Jigna Patel

**Affiliations:** 1 Rutgers University, Newark, New Jersey, USA

No further comments to make.

Is the work clearly and accurately presented and does it cite the current literature?

Yes

If applicable, is the statistical analysis and its interpretation appropriate?

Yes

Are all the source data underlying the results available to ensure full reproducibility?

Yes

Is the study design appropriate and is the work technically sound?

Partly

Are the conclusions drawn adequately supported by the results?

Yes

Are sufficient details of methods and analysis provided to allow replication by others?

Yes

Reviewer Expertise:

Neurorehabilitation and neuroscience and cardiopulmonary PT.

I confirm that I have read this submission and believe that I have an appropriate level of expertise to confirm that it is of an acceptable scientific standard.

# Reviewer report: Reviewer response for version 2

**DOI:** 10.5256/f1000research.158690.r222342

**Published:** 2023-11-10

**Authors:** Ketaki Inamdar

**Affiliations:** 1 Virginia Commonwealth University, Richmond, Virginia, USA

The authors have done a commendable job in incorporating my feedback. The revised version has a more robust introduction and discussion section, with more balanced conclusions. Here are some minor suggestions for the authors.

1. Consider rephrasing the last line of the first paragraph in the Introduction section; it appears to contain a minor grammatical error.
2. Given the exclusion of the multi-rater kappa analysis for different time-points, kindly remove it from the analysis plan.

Is the work clearly and accurately presented and does it cite the current literature?

Yes

If applicable, is the statistical analysis and its interpretation appropriate?

Partly

Are all the source data underlying the results available to ensure full reproducibility?

Yes

Is the study design appropriate and is the work technically sound?

Partly

Are the conclusions drawn adequately supported by the results?

Partly

Are sufficient details of methods and analysis provided to allow replication by others?

Yes

Reviewer Expertise:

NA

I confirm that I have read this submission and believe that I have an appropriate level of expertise to confirm that it is of an acceptable scientific standard.

# Reviewer report: Reviewer response for version 1

**DOI:** 10.5256/f1000research.136897.r208335

**Published:** 2023-10-12

**Authors:** Ketaki Inamdar

**Affiliations:** 1 Virginia Commonwealth University, Richmond, Virginia, USA

It was a pleasure to read the paper. The study's results tackle a persistent challenge in literature, namely the monitoring of physical and other activities in clinical populations, offering a novel solution *. For this reason, I believe these findings should be considered for indexing*. However, I would like to recommend significant modifications to enhance the scientific rigor of the study. The current results are presented too strongly given the design and sample size. I suggest toning down the findings, emphasizing the pilot and feasibility testing component of the study, and engaging in a critical discussion on how these results can inform the design of caregiver-monitored activity logs during early-phase stroke rehabilitation.

**Title:**

- Consider adding "Pilot" to the title due to the sample size.

**Abstract:**

 Well-written, minor suggestions:

- Consider adding "early phase" before "hospital settings" to distinguish from outpatient rehabilitation.
- Insert "across one day" before "every 15 minutes" for clarity.
- Include brief demographics for physiotherapists as participants.

**Introduction:**

 Paragraph 1:

 Please incorporate stroke statistics specific to India to substantiate the study's relevance. Utilize percentages to illustrate the impact, particularly when stating that "stroke survivors have significantly reduced activity levels."

- Define "early phase" at the outset to enhance the paper's readability.

Paragraph 2:

- Expand on the benefits of increased activity during the acute phase of stroke recovery and delineate the factors that limit activity, especially in the post-acute phase.

Paragraph 3:

- Briefly talk about the different tools used for behavioural mapping such as video recording, checklists, wearable devices etc., with a brief discussion on pros and cons for each method. Please justify why you selected checklist/log.

**Methods:**

*Inclusion criteria: *

- “Functionally communicate” has been used twice in the paragraph. 
- Include the inclusion criteria for physiotherapists, specifying their level of expertise (e.g., trained neurophysiotherapists, novice clinicians, experienced clinicians) to provide context on their participation and qualifications in the study.

*Outcome*:

- Elaborate on the process of developing the activity log, specifying whether the validation involved face validation or content validation, to enhance the clarity and rigor of the log development.
- Specify whether the testing involving 20 stroke patients was related to assessing the practicality and usability of the log. This clarification will provide a better understanding of the purpose and focus of the testing phase.
- Finally, explain why exercise was categorized separately from therapies in the activity log. Given that therapies often encompass exercise in the early phase, clarify the rationale for this distinction and consider discussing how this categorization may have contributed to any discrepancies observed between physiotherapists and caregivers.

**Procedure:**

- Clarify whether the intern-to-patient ratio was 1:1 or if the intern monitored more than one patient on a particular day. Specify the procedure for patient monitoring when the intern had to take a break.
- Indicate whether caregivers were provided with reminders or prompts every 15 minutes during the monitoring process.
- Provide a rationale for selecting only one working day for agreement testing.

**Statistical Analysis:**

- Due to the limited sample size in this study, it is important to provide a justification for the chosen sample size. Include information regarding whether a power analysis was conducted.
- I believe the study lacks sufficient statistical power for utilizing multi-rater kappa, a concern that is indeed emphasized in the discussion. It might be worth considering excluding these specific findings.

**Results:**

- Integrate relevant details about the physiotherapists into the tables or corresponding paragraphs within the Results section.
- Organizing the kappa agreement according to the three domains of activities in Figure 1 would be beneficial. This would offer insights into the level of agreement within each domain, allowing for a comparison of agreement quality across the domains.

**Discussion:**

 Overall, the authors effectively emphasize the feasibility of utilizing caregiver led activity logs and underscore how the design of the activity log can impact agreement. However, enhancing the discussion through a more critical comparison with existing studies would further bolster the strength of the argument.

 Here are some suggestions for the discussion:

- Draw parallels with the fundamental concepts of Ecological Momentary Assessment (EMA) and elaborate on how periodic reminders every 15 minutes could have impacted the reporting within this study.
- Compare the role of caregiver burden and recall burden in subjective reporting studies conducted in similar and other clinical populations.
- Address the learning curve associated with reporting multiple activities by caregivers. The limited reporting duration in this single-day study may not have allowed caregivers to fully grasp the intricacies of reporting various activities, especially if a change in caregivers occurred during the day.
- A discussion on alternative activity tracking methods, such as wearable sensors, should have been included.
- Include a discussion of the rationale behind selecting specific activities, particularly during the initial phase, and its influence on the reporting process. For instance, considering that physiotherapists were unable to access the bathroom area, consolidating toileting-related activities into a single category could ensure equal reporting opportunities for both physiotherapists and caregivers. Likewise, it is anticipated that patients would undergo clinical monitoring and testing early in their recovery phase. Future studies may consider incorporating this aspect into the activity log to mitigate discrepancies related to this particular activity.

Is the work clearly and accurately presented and does it cite the current literature?

Yes

If applicable, is the statistical analysis and its interpretation appropriate?

Partly

Are all the source data underlying the results available to ensure full reproducibility?

Yes

Is the study design appropriate and is the work technically sound?

Partly

Are the conclusions drawn adequately supported by the results?

Partly

Are sufficient details of methods and analysis provided to allow replication by others?

Yes

Reviewer Expertise:

Neurophysiotherapy, activity monitoring

I confirm that I have read this submission and believe that I have an appropriate level of expertise to confirm that it is of an acceptable scientific standard, however I have significant reservations, as outlined above.

# Response

**Published:** 2023-11-07

**Authors:** John M Solomon

**Affiliations:** Manipal Academy of Higher Education, India

We, the authors, convey our sincere thanks to the reviewer for spending their valuable time reviewing our manuscript. We are pleased to know the suggestions/ modifications advised. The suggestions helped to enhance the quality of the manuscript. All the suggestions were incorporated/ addressed in the revised manuscript. Our response can be identified below in italics and bold.

1. Title: Consider adding "Pilot" to the title due to the sample size.

***Thank you, we agree. We have made a request to the journal for a change in title.***

  

1. Abstract: Well-written, minor suggestions:

•              Consider adding "early phase" before "hospital settings" to distinguish from outpatient rehabilitation.

 •              Insert "across one day" before "every 15 minutes" for clarity

 •              Include brief demographics for physiotherapists as participants.

***The authors would like to thank the reviewer for the suggestion. The suggested changes have been incorporated into the abstract.***

  

1. Introduction:

Paragraph 1: Please incorporate stroke statistics specific to India to substantiate the study's relevance. Utilize percentages to illustrate the impact, particularly when stating that "stroke survivors have significantly reduced activity levels."

 •              Define "early phase" at the outset to enhance the paper's readability.

 Paragraph 2: Expand on the benefits of increased activity during the acute phase of stroke recovery and delineate the factors that limit activity, especially in the post-acute phase.

 Paragraph 3: Briefly talk about the different tools used for behavioural mapping such as video recording, checklists, wearable devices etc., with a brief discussion on pros and cons for each method. Please justify why you selected checklist/log.

***Thank you for this suggestion. We have added the prevalence data from India and the percentage to the statement. This brings in more clarity to the given information.***

***Early phase has been defined as suggested. Thank you***

***We appreciate this suggestion to emphasize the importance of being active and the same has been added.***

***We agree that the details would give a better insight. We have added more information about the different tools used.***

  

1. Methods:

Inclusion criteria:

 “Functionally communicate” has been used twice in the paragraph. 

 Include the inclusion criteria for physiotherapists, specifying their level of expertise (e.g., trained neurophysiotherapists, novice clinicians, experienced clinicians) to provide context on their participation and qualifications in the study.

***Thank you for the suggestion. We have used it twice for two different population. The first one refers to the eligibility in stroke survivors and the second mentioning refers to their caregivers.***

***We are obliged to rectify this. The same has been added in the methods section.***

  

1. Outcome:

•              Elaborate on the process of developing the activity log, specifying whether the validation involved face validation or content validation, to enhance the clarity and rigor of the log development.

 •              Specify whether the testing involving 20 stroke patients was related to assessing the practicality and usability of the log. This clarification will provide a better understanding of the purpose and focus of the testing phase.

 •              Finally, explain why exercise was categorized separately from therapies in the activity log. Given that therapies often encompass exercise in the early phase, clarify the rationale for this distinction and consider discussing how this categorization may have contributed to any discrepancies observed between physiotherapists and caregivers.

***The author would like to thank the reviewer for these suggestions. We have added content validation of the log chart to bring in more clarity and the objective of the testing.***

***We appreciate the suggestion and have added the same. However, in our opinion, these details would better suit in the manuscript pertaining to the development of the activity log chart.***

  

1. Procedure:

•              Clarify whether the intern-to-patient ratio was 1:1 or if the intern monitored more than one patient on a particular day. Specify the procedure for patient monitoring when the intern had to take a break.

 •              Indicate whether caregivers were provided with reminders or prompts every 15 minutes during the monitoring process.

 •              Provide a rationale for selecting only one working day for agreement testing.

***Thank you for the valuable suggestion. This will help give more details about the procedure. We have edited the same.***

***We have added the suggested information. Thank you.***

***We are obliged to rectify this. The same has been added.***

  

1. Statistical Analysis:

•              Due to the limited sample size in this study, it is important to provide a justification for the chosen sample size. Include information regarding whether a power analysis was conducted.

 •              I believe the study lacks sufficient statistical power for utilizing multi-rater kappa, a concern that is indeed emphasized in the discussion. It might be worth considering excluding these specific findings.

***We agree with the reviewer. We did a power analysis and the same has been noted in the statistics and results part of the paper. We did not calculate the sample size as this was a pilot study.***

***We are obliged to rectify this and have removed the same from the manuscript.***

  

1. Results:

•              Integrate relevant details about the physiotherapists into the tables or corresponding paragraphs within the Results section.

 •              Organizing the kappa agreement according to the three domains of activities in Figure 1 would be beneficial. This would offer insights into the level of agreement within each domain, allowing for a comparison of agreement quality across the domains.

***We are obliged to rectify this. The same has been added in the text of the results section.***

***Thank you for the suggestion. In our opinion, arranging the items according to the descending order brings in readability and clarity. However, the suggested information has already been added in the text section.***

1. Discussion:

Overall, the authors effectively emphasize the feasibility of utilizing caregiver led activity logs and underscore how the design of the activity log can impact agreement. However, enhancing the discussion through a more critical comparison with existing studies would further bolster the strength of the argument.Here are some suggestions for the discussion:

 •              Draw parallels with the fundamental concepts of Ecological Momentary Assessment (EMA) and elaborate on how periodic reminders every 15 minutes could have impacted the reporting within this study.

 •              Compare the role of caregiver burden and recall burden in subjective reporting studies conducted in similar and other clinical populations.

 •              Address the learning curve associated with reporting multiple activities by caregivers. The limited reporting duration in this single-day study may not have allowed caregivers to fully grasp the intricacies of reporting various activities, especially if a change in caregivers occurred during the day.

 •              A discussion on alternative activity tracking methods, such as wearable sensors, should have been included.

 •              Include a discussion of the rationale behind selecting specific activities, particularly during the initial phase, and its influence on the reporting process. For instance, considering that physiotherapists were unable to access the bathroom area, consolidating toileting-related activities into a single category could ensure equal reporting opportunities for both physiotherapists and caregivers. Likewise, it is anticipated that patients would undergo clinical monitoring and testing early in their recovery phase. Future studies may consider incorporating this aspect into the activity log to mitigate discrepancies related to this particular activity.

***Thank you. We appreciate the suggestions put forth for our discussion.***

***This is a very good point to add. The authors appreciate the reviewer for the suggestion. We have added details of the same.***

***Thank you for the suggestion. The role of caregiver and the burden has already been discussed.***

***We agree that this would be an important finding to discuss. The same has been added.***

***Thank you. We agree to this. We have added the same in the discussion.***

***We appreciate the detailed suggestion. This is very insightful. We have added the same.***

# Reviewer report: Reviewer response for version 1

**DOI:** 10.5256/f1000research.136897.r188815

**Published:** 2023-08-04

**Authors:** Shuanglan Lin

**Affiliations:** 1 Chongqing Medical University, Chongqing, China

***Summary***

 Thank you for the invitation to review this paper. The paper might provide evidence about an alternative option to address activity monitoring in the clinical setting by caregivers (especially in some activities such as bathing and dressing), as this could be time-consuming for healthcare professionals. However, the research methodology and the small sample size in the paper might result in a lack of credibility and validation of the findings. The details of the comments are listed as follows:

***Introduction***

1. Please provide more information about the stroke status and stroke survivors in India since the study was conducted there.

***Eligibility criteria***

1. Please list the inclusion and exclusion criteria for the participants to help readers better understand this section.

***Outcome***

1. Please clarify the specific activities included in the physical, cognitive and social components.
2. Please describe the log chart’s validation process and provide more information about the experts.

***Procedure ***

1. Please clarify if the caregivers and physiotherapists received the same training about using the activity log chart to ensure activity marking consistency during the implementation.
2. A single-day observation of activities among 17 stroke survivors might not provide enough evidence and draw a solid conclusion for the study. Plus, behavioural mapping is a subjective process that can lead to variability during observations.

***Results ***

1. In Figure 1, it’s better to categorise all the recorded activities under the three components to help readers better understand “ *the agreement of activities under physical, cognitive and social domains were 42, 38 and 43%, respectively*”.
2. I suggest analysing the factors that cause the inconsistency of agreement between caregivers and the physiotherapist, such as the physiotherapist who have received professional training and better understands different activities, or whether the caregivers’ educational background will lead to the deviation of observation.

***Discussion ***

1. “ *Both the physiotherapist and the caregiver did not complete all observations*” implies that the study’s observations were done only on a single day is not enough to conclude.
2. As some of the toiletry activities were monitored only by the caregivers, there is still a potential for caregivers to perform behavioural mapping of stroke in the clinical after clarifying and addressing the factors that caused the discrepancy of agreement between caregivers and the physiotherapist.

Is the work clearly and accurately presented and does it cite the current literature?

Partly

If applicable, is the statistical analysis and its interpretation appropriate?

Yes

Are all the source data underlying the results available to ensure full reproducibility?

Yes

Is the study design appropriate and is the work technically sound?

No

Are the conclusions drawn adequately supported by the results?

Partly

Are sufficient details of methods and analysis provided to allow replication by others?

Partly

Reviewer Expertise:

Stroke, stroke transitional care, data analysis

I confirm that I have read this submission and believe that I have an appropriate level of expertise to state that I do not consider it to be of an acceptable scientific standard, for reasons outlined above.

# Reviewer report: Reviewer response for version 1

**DOI:** 10.5256/f1000research.136897.r188821

**Published:** 2023-08-03

**Authors:** Jigna Patel

**Affiliations:** 1 Rutgers University, Newark, New Jersey, USA

This is a simple study design that asks an important question. Specifically, the authors compared the activity monitoring of stroke survivors by caregivers and physiotherapists in a hospital setting. The amount of activity performed by stroke survivors early after stroke may affect long term recovery, thus activity monitoring by caregivers can provide an alternate means of tracking this measure. The authors found a 60% agreement between the physiotherapists and the caregivers, using Kappa statistics. The study design has some limitations which are explained by the authors. 

 Minor comments: 

1. The second sentence in second paragraph of introduction is awkward. Are you trying to say there is evidence that recovery happens rapidly in the acute phase? Please reword. 
2. Last sentence in introduction should say amount of 'activity' not 'activities'. 
3. Remove the word 'By' at the beginning of the first sentence in the fourth paragraph of introduction. 
4. In the eligibility section, you use the word 'supratentorial', so only cortical strokes included? Please clarify. 
5. Procedure section: change sentences to, 'patients and caregivers were given explanations about the study'. 

I approve this study as is, with the changes noted in the minor comments section.

Is the work clearly and accurately presented and does it cite the current literature?

Yes

If applicable, is the statistical analysis and its interpretation appropriate?

Yes

Are all the source data underlying the results available to ensure full reproducibility?

Yes

Is the study design appropriate and is the work technically sound?

Partly

Are the conclusions drawn adequately supported by the results?

Yes

Are sufficient details of methods and analysis provided to allow replication by others?

Yes

Reviewer Expertise:

Neurorehabilitation and neuroscience and cardiopulmonary PT.

I confirm that I have read this submission and believe that I have an appropriate level of expertise to confirm that it is of an acceptable scientific standard.

# Reviewer report: Reviewer response for version 1

**DOI:** 10.5256/f1000research.136897.r170683

**Published:** 2023-06-01

**Authors:** Lucian Bezuidenhout

**Affiliations:** 1 Department of Neurobiology, Care Sciences and Society, Division of Physiotherapy, Karolinska Institutet, Stockholm, Sweden

**Summary**

 Thank you for the opportunity to review this paper. Overall, the paper tackles an interesting topic, which is comparing the activity monitoring of stroke survivors by physiotherapists and caregivers. While this is a relevant topic and has some merit since caregiver monitoring can potentially be cost-effective and time efficient, however, there are some major concerns that I would like the authors to clarify. More specifically, regarding the methods used. The authors only used 1 day, although I don’t agree with 1 day being a valid measurement period for 17 stroke survivors. The authors need to strongly justify this, especially due to the subjectivity of rating by both the physios and caregivers and the experience of the caregivers in using the rating tool. In addition to this, there is missing data on observing (i.e. toileting, bathing, testing). The hypothesis of the authors also assumes that caregivers will be there to monitor the patients all day around, which is not always possible.

**Abstract**

- The first line of the conclusion " *There was moderate agreement between the physiotherapist and caregiver for activity monitoring of stroke survivors*" is rather a result than a conclusion. I suggest the authors rephrase the conclusion to conclude the overall outcome of their study.

**Introduction**

- In the first paragraph, the authors write about the global and European prevalence of stroke. While this is good, I suggest the authors also add about the prevalence of stroke in India, since this is where the study was conducted.
- In the second paragraph, the authors address the physical (in)activity of people with stroke during inpatient rehab. Are there any recommendations for PA during this period for stroke survivors? i.e. number of steps per day or min spent in different PA levels for example?
- In the third paragraph, the authors briefly touch on the behavioral mapping tool. I think this paragraph could be strengthened by expanding on this tool, i.e., what does it consist of? Also, the authors need to address previous studies that have used this tool in people with stroke, what have they found? Are there previous studies that have done something like your study?

**Methods**

**In the 'Eligibility criteria' paragraph:**

- Can the authors give examples of who the caregivers could be?
- Also, can the authors first list all the inclusion criteria and then list the exclusion criteria? Was understanding or previous experience of the activity log an inclusion criterion?

**In the 'Outcome' paragraph:**

- Did the caregivers or the physios get any training on using the activity log?
- The authors mention that " *the log chart was sent to 15 experts for validation*". How was this done? Who were the experts?

**Procedure**

- The authors mention that " *The observations were carried out every 15 min*" *. *What happened when the caregivers or physios had to take a break i.e. go to the toilet or lunch break?
- Is one day enough to make an overall conclusion? The authors need to strongly justify why they only did 1 day of measurements.

**Results**

- Figure 1, can the authors separate (group) the different activities into the 3 different domains i.e. physical, cognitive and social? Therefore, it would be easier to see which of these domains had the best agreement or the worst.
- It would also be interesting to look at the agreement over the course of the day. For example, did the accuracy decrease over the day i.e., did the raters get tired of observing the people with stroke?

**Discussion**

- The discussion lacks comparison to other studies that conducted similar observations. What did they find compared to your results?
- Have the authors thought about using accelerometry to measure activity? Although the accelerometry might not be able to yield the exact activity being done, it could give an overall PA output, which could be a good marker for measuring changes in activity levels. I think this is worth a mention in the discussion.
- Although I think one can't really draw any conclusion from 1 day of observations, what does it mean clinically that there is a 60% agreement between the physiotherapist and the caregivers? Is this enough? What can be done to increase this agreement? Is this a future valid technique?
- One of the possible clinical implications of this study is if the caregivers don't want to participate in the activity logging. Maybe they just want to be present and support the person affected by stroke.  
- This paper does not have an overall conclusion.

Is the work clearly and accurately presented and does it cite the current literature?

Partly

If applicable, is the statistical analysis and its interpretation appropriate?

Yes

Are all the source data underlying the results available to ensure full reproducibility?

Yes

Is the study design appropriate and is the work technically sound?

No

Are the conclusions drawn adequately supported by the results?

No

Are sufficient details of methods and analysis provided to allow replication by others?

Partly

Reviewer Expertise:

Physical activity, Stroke, neurological diseases. Data analysis procedures, scientific methods.

I confirm that I have read this submission and believe that I have an appropriate level of expertise to state that I do not consider it to be of an acceptable scientific standard, for reasons outlined above.
